# Surface-Enhanced Raman Scattering-Based Lateral-Flow Immunoassay

**DOI:** 10.3390/nano10112228

**Published:** 2020-11-10

**Authors:** Boris Khlebtsov, Nikolai Khlebtsov

**Affiliations:** 1 Institute of Biochemistry and Physiology of Plants and Microorganisms, Russian Academy of Sciences, 410049 Saratov, Russia; khlebtsov_n@ibppm.ru; 2 Faculty of Nano- and Biomedical Technologies, Saratov State University, 410012 Saratov, Russia

**Keywords:** surface-enhanced Raman scattering, lateral flow immunoassay, tags, immunochromatography

## Abstract

Lateral flow immunoassays (LFIAs) have been developed and used in a wide range of applications, in point-of-care disease diagnoses, environmental safety, and food control. However, in its classical version, it has low sensitivity and can only perform semiquantitative detection, based on colorimetric signals. Over the past decade, surface-enhanced Raman scattering (SERS) tags have been developed in order to decrease the detection limit and enable the quantitative analysis of analytes. Of note, these tags needed new readout systems and signal processing algorithms, while the LFIA design remained unchanged. This review highlights SERS strategies of signal enhancement for LFIAs. The types of labels used, the possible gain in sensitivity from their use, methods of reading and processing the signal, and the prospects for use are discussed.

## 1. Introduction

**Figure 1:**
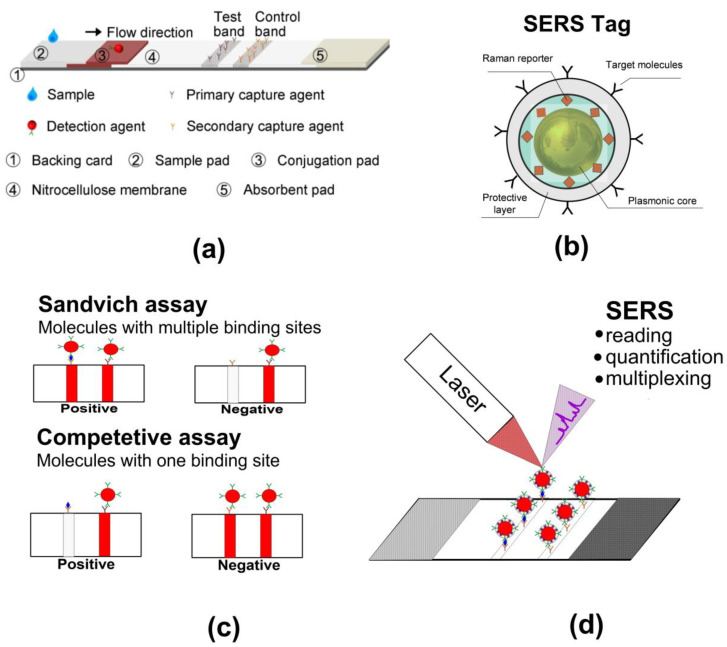
(**a**) Schematics showing the (A) working principle and components of LFIA. Reproduced with permission from [8]. (**b**) General scheme of SERS tag composition. (**c**) Schematic representation of sandwich and competitive lateral flow immunoassays (LFIA) results. (**d**) Schematic representation of surface-enhanced Raman scattering (SERS) LFIA experiment.

Lateral flow immunoassay emerged as a simple and inexpensive method for diagnosis at the point of care in the early 1990s [1,2]. Compared to the other immunological methods, such as using enzyme-linked immunoassay (ELISA), LFIAs require far less sample preparation and signal readout time [3]. A typical LFIA test system is a paper-based unit, which consists of five main components: a backing card, a nitrocellulose membrane, a sample pad, a conjugate pad, and an absorbent pad (Figure 1a). Additionally, further required components comprise three types of antibodies and colloidal labels. One type of monoclonal antibody is applied to the membrane as a line or spot in the test (T) zone, the second type of monoclonal antibody is conjugated to the labels, and polyclonal antibodies, specific to the monoclonal antibodies, are applied to the control (C) zone. The assay can be initiated by applying the analyte to the sample pad and does not require additional reagents and processing steps. The analyte of interest moves by capillary flow, together with the immunocomplex, along the membrane and interacts with antibodies in the control and test zones. As a result, the colored zones occur and further analyses are performed by the naked eye or through optical techniques [3,4]. Depending on the type of antigen being analyzed, the analysis is carried out in different formats. A sandwich format is used to detect relatively large molecules (e.g., proteins) in a sample. In this case, two clones of monoclonal antibodies specific to different fragments of the analyte are used. As a result, a positive test result appears as two colored areas on the nitrocellulose membrane, and a negative result appears as one colored area (Figure 1c). If the analyte molecule is small and has only one binding site, then it is advisable to use a competitive immunoassay. In this case, there is a competitive binding of antibodies with analyte molecules or other antibodies. A positive test result manifests one colored line, and a negative one, two lines (Figure 1c). A wide variety of labels have been proposed for use in the LFIA, including gold nanoparticles, quantum dots, magnetic particles, colloidal selenium, radioactive isotopes, and many others [4,5,6,7,8]. Despite this diversity, gold nanoparticles are the most commonly used labels in LFIA as a result of their ease of fabrication, functionalization, and strong light extinction [9,10]. Localized surface plasmon resonance (LSPR) provides the bright red color of colloidal gold and thus the color of areas with adsorbed particles. Another obvious advantage of colloidal gold is that it can be used to visualize test results (in terms of positive and negative) without the use of any equipment. If there are enough particles in the test zone, its color is visible to the naked eye. We recently estimated the required particle densities, depending on their size. So, for particles with a size of 16 nm, it is necessary that the biospecific reaction delivers at least 6.7 × 10^7^ particles/mm^2^ to the test zone [11]. This substantial surface density explains one of the first drawbacks of LFIA—low sensitivity relative to other immunological methods.

For some applications, such as detecting pregnancy or the presence of traces of drugs in body fluids, a yes/no test is sufficient. However, there are a number of analytical applications where it is necessary to quantify the concentration of an analyte. Because a change in analyte concentration leads to a change in the concentration of labels in the test zone, it can be measured by the colorimetry of test zone. In the simplest case, this can be done by scanning the membrane with a conventional scanner and assessing the brightness of the colors in the test zone. There are also a number of commercial instruments available for converting LFIA results to numerical format from colorimetric results [12]. Occasionally, even a smartphone camera and a special application can be used for this [13,14]. It should be noted, however, that the working range of analyte concentrations for colorimetric detection is usually too narrow for successful applications. For concentrations that are too low, the number of particles in the test zone is insufficient to form a colored band, and for high concentrations, color saturation quickly occurs. Even in the working range, the dependence of the color saturation of the test zone on the concentration of the analyte is highly nonlinear (usually logarithmic). In this regard, the search for new types of labels and optical readout technologies is an urgent task for expanding the directions of applications of LFIA.

Surface-enhanced Raman scattering (SERS) tags have received much attention in various analytical and imaging applications [15]. SERS tags consist of Au nanoparticles, serving as a plasmonic core, Raman-active molecules adsorbed or embedded in nanoparticles, and a protective polymer or inorganic layer, which is functionalized with recognizing antibodies (Figure 1b) [16]. SERS tags can have a much higher optical efficiency than that for fluorescent labels, such as organic dyes and quantum dots [15]. From a physicochemical point of view, SERS tags resemble simple colloidal gold, thus we can use them in the same LFIA design (Figure 1d). On the other hand, from an optical point of view, SERS tags have several attractive advantages. First, the intensity of enhanced Raman scattering makes it possible to detect even a single SERS tag using a common optical technique [17]. Second, the intensity of the SERS signal is proportional to the tag number in a wide range of concentrations. Finally, the narrow spectral bands of Raman scattering make it suitable for multiplexing. This has led to the emergence of a number of studies demonstrating significant increases in the sensitivity and dynamic range of LFIA using the SERS readout.

Hence, in this review, we try to cover SERS-based approaches in lateral flow immunoassay. Specifically, we start with the main types of SERS tags used in LFIA (Section 2). Then, we focus on the devices and algorithms used for signal measurement, averaging, and baseline correction (Section 3). In Section 4, we describe the obtained decrease in the limit of detection, together with a discussion of the mechanisms of this improvement. In Section 5, we discuss the possibility of multiplexing. Finally, the challenges and future perspectives of SERS-LFIA are presented in the conclusion section.

## 2. SERS Tags for LFIA

**Figure 2:**
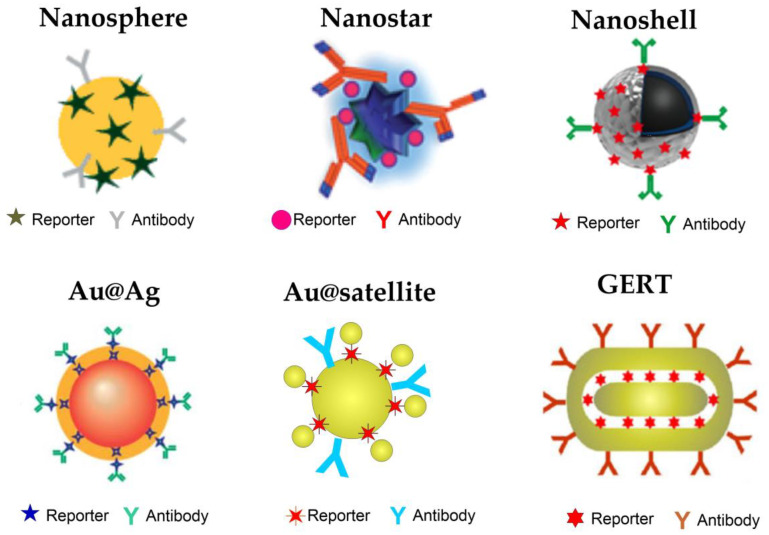
A gallery of SERS tags used for LFIA. The gallery includes Au nanospheres, nanostars, nanoshells, Au@Ag core/shell particles, Au@satellites, and gap-enhanced Raman tags (GERTs).

The use of SERS tags for LFIA has been explored as a novel assay platform since 2007 [18]. Because the SERS tags determine the specificity and quantitative manner of detection, their structure should be designed to improve the performance of assays (Figure 2). Generally, the aim for designing SERS tags is to improve sensitivity, chemical and physical stability, as well as their multiplexing ability. A typical SERS tag contains four components: a plasmonic nanoparticle, a Raman reporter molecule, a protection layer, and an antibody. To obtain a large enhancement factor and high stability of Raman signals, the structure of metal nanoparticles needs to be carefully designed. The first example of SERS LFIA was performed using a simple spherical Au nanoparticle [18]. In general, this type of particle seems to be a suitable candidate for the SERS assay because of its easy synthesis and conjugation, and many papers have reported their successful application [19,20,21,22,23,24,25,26,27,28,29,30,31,32,33]. However, SERS response from Au nanospheres is quite small. The typical fundamental enhancement factor for Au nanospheres is about 10^3^–10^4^ depending on size and laser wavelength. This value is much lower than that reported for optimal nanotags reaching 10^8^ or higher [15,34]. Using Ag nanoparticles instead of Au can improve the SERS response, and one article reports the use of Ag spheres as SERS tags for LFIA [35]. The main disadvantages of Ag nanoparticles are related to their polydispersity and instability in buffers. In recent decades, metal nanoparticles with different shapes, including nanoshells, nanorods, nanostars, and core/shell metal particles, have been suggested. Because of the core/shell structure and tunable plasmon resonance, hollow Au nanoshells [36] have been used as effective SERS tags for LFIA detection and the quantification of Staphylococcal enterotoxin B. Additional advantages related to the magnetic purification of the samples have been demonstrated using Au nanoshells on a magnetic core [37,38]. Nanoparticles with sharp tips and nanoscale gaps have a large number of hot spots, resulting in a higher SERS enhancement factor, compared to that of the nanospheres. The examples of branched particles as SERS tags for LFIA include nanostars [39,40,41], nanoflowers [42], raspberry-like particles [43], and composite Au@PT rough nanorods [44]. Moreover, high EF branched metal nanoparticles have attracted much attention due to increased surface-to-volume ratios, allowing for more Raman reporters to attach to their surfaces. On the other hand, such particles usually have quite large size—about 70–100 nm. This, together with their rough surface, decrease their colloidal stability and make uniform movement through the membrane under lateral flow challenging.

Recently, core–shell-type metal nanoparticles have also been widely used for designing SERS probes. By combining the high stability and monodispersity of gold and the large enhancement factor of silver, Au@Ag [27,45,46,47,48,49,50,51], Ag@Au [52,53,54], or bimetallic [55,56], SERS probes can be designed. For this, particle Raman reporters can not only be adsorbed onto the nanoparticle surface but also embedded between Au and Ag layers. Recently, we have demonstrated that such a position of reporters leads to higher SERS response from the core/shell particle [57]. Additionally, the outer metal layer serves both as a signal amplifier and as a protecting agent. Thus, molecules inside the core/shell particles are protected from desorption and the nanoparticle surface is ready for conjugation. In addition to selecting metal nanoparticles with different shapes, another important method to improve SERS activity is to create “hot spots” through controlled gap formation. Several groups have reported the fabrication of SERS tags for LFIA using Au core@ satellite nanoparticles [58,59]. Another possible approach is the formation of nanoparticles containing interior nanogaps [60]. These new types of SERS nanoprobes, named “gap-enhanced Raman tags (GERTs),” have been reported to show outstanding Raman enhancement, reproducibility, and photostability. Such types of particles have recently been reported as SERS LFIA for the quantitative detection of cardiac troponin I [61] and hCG [62].

The second obligatory component after the plasmon core is the Raman reporter. The ideal Raman reporter should meet several important criteria. The first is a large Raman cross-section. The second is the possibility of obtaining a conjugate with a plasmonic core by simple mixing. The third is the stability to photobleaching. Finally, a reporter molecule should promote further conjugation of the SERS tag to antibodies. There are two main types of molecular agents used as Raman reporters for the synthesis of SERS tags for LFIA: dye molecules and thiolated aromatic molecules. The most popular among the dyes is malachite green isothiocyanate (MHITC) [19,23,24,36]. It is a well-known reporter with quite a large cross-section and numerous Raman peaks in the range of 400–1600 cm^−1^. Conjugation is usually carried out by simple physical adsorption of the dye to the surface of the particle over several hours of incubation. There are two papers that used Nile blue dye (NBA) as the Raman reporter [48,56]. The adsorption of dye molecules on the nanoparticle surface typically did not totally block it. Thus, further conjugation tags with antibodies were carried out by simple physical adsorption. Thiolated aromatic molecules are also very popular Raman reporters because of the high affinity of the thiol group to Au or Ag and the well-defined Raman peaks of benzene ring vibration. The most popular Reporters are mercaptobenzoic acid [21,40,42,43,44,46,47] and dithionitrobenzoic acid [22,27,29,30,38,43,49]. These molecules have a carboxyl group, which can promote conjugation to antibodies using traditional EDC/NHS cross-linking. The other examples of thiolated aromatic molecules as Raman reporters include aminothiophenol [39,41,55], benzene dithiol [62], naphthalene thiol [59], and nitrobenzene thiol [61]. To improve the colloidal stability of SERS tags, as a rule, the same approach is used as for traditional LFIA labels, namely, the addition of bovine serum albumin as a secondary stabilizer after conjugation. Another possible approach is to coat the particle with a silica layer [21,23,62].

## 3. SERS Signal Accumulation and Data Processing

The methodology of SERS-based LFIA is generally the same as for conventional LFIA at the steps of strip construction and sample application. The main differences derive from the steps of signal readout and processing. In the SERS-based LFIA strip, antibody-conjugated SERS tags are used as probes instead of antibody-conjugated AuNPs used in the conventional LFIA strip. Thus, SERS tags accumulated in control and test zones make them Raman active.

**Figure 3:**
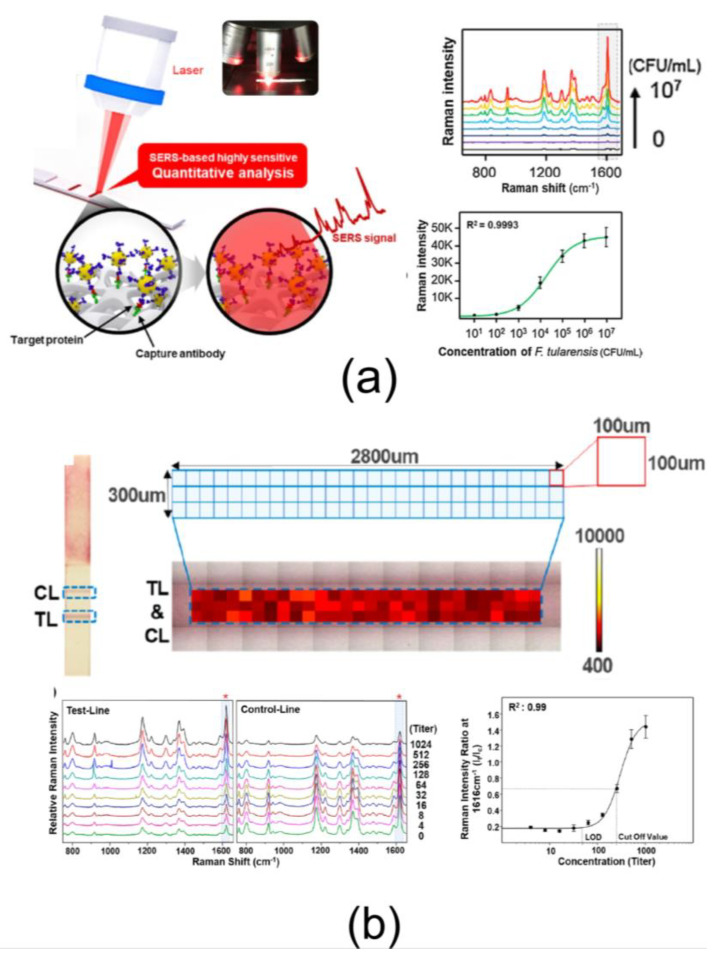
(**a**) Using the SERS-based LFA strip and measuring the signal in separated points of T line quantitative analysis of a specific target can be performed. Quantitative SERS-based assay results for bacterial pathogen *F. tularensis*. The assays were performed in the 0–10^7^ CFU/mL range and corresponding calibration curves for different concentrations of the bacterial pathogen. Reproduced with permission from [31]. (**b**) Schematic illustration of the Raman data collection process. The mapping area for the test and control lines. A total of 84 pixels were imaged with 100 μm intervals. Average SERS spectra of 84 pixel points for different titer concentrations of *O. tsutsugamushi*-specific IgG antibodies on the test and control lines. Corresponding standard calibration curve for different titer concentrations of the antibodies. Reproduced with permission from [24].

The following task is to read and quantify the SERS signal from the control (C) and test (T) zones (or bands) (see Figure 1). The common devises for SERS reading consist of a laser, an objective lens for focusing light on a substrate, and a spectrometer to collect the signal. In the simplest way, the SERS signal can be acquired and averaged from several independent points in the test and control zones (Figure 3a). Typically, in the course of calibration experiments, the SERS spectra in the test zone are measured for various analyte concentrations. Then, the required spectral range is selected, and a calibration curve is constructed from which important parameters, such as the detection limit and the working concentration range are determined. The approach, based on measuring and averaging the signal at several points within the T and C zones, has been successfully applied in many papers for cardio biomarker detection and quantification [56], HIV DNA detection [20], detection of neuron-specific enolase in blood plasma [21], detection of the β-adrenergic agonist brombuterol [42], and many other applications [25,35,39,40,41,43,44,46,47,55,63,64,65]. However, a number of questions arise regarding the implementation of this approach. First, how many points within the zone must be selected to obtain a reliable result? How should you choose these points, as the distribution of particles can be very uneven? What is the correct way to maintain the same focus of laser radiation on these points? Indeed, the reproducibility of the Raman measurements is critical in SERS-based immunoassays. In many cases, the measurement of the Raman signal is carried out using a Raman microscope. As a rule, this device has the ability to construct a Raman map—i.e., the dependence of the Raman signal on each point of the selected area. From this point of view, Raman casing of the entire T and C zones on the membrane, followed by averaging of SERS spectra, looks promising. For example, Lee et al. [24] demonstrate full T and C zone Raman mapping for quantitative evaluation of the *O. tsutsugamushi*-specific IgG antibody. Figure 3a illustrates the spot-to-spot Raman detection process for the test and control lines. To identify a Raman mapping area on an assay strip, the laser spot was scanned in the direction perpendicular to the test line. The corresponding mapping area covers all T or C zones. The Raman mapping image was acquired using 100 μm mapping intervals for each laser spot. This resulted in a total of 84 mapping spectra being obtained in 42 s. Raman mapping clearly demonstrates large signal variations from point to point, which further emphasizes the need for large area mapping rather than multipoint measurement. Figure 3b shows the average Raman spectra of the 84 pixel points for the test (left) and control (right) lines for various titer concentrations. Using this strip, the presence of *O. tsutsugamushi*-specific IgG antibodies can be determined through the color change of the test line. Quantitative analysis of the antibodies can also be achieved by monitoring their Raman intensity. With increasing titer concentration, there is an obvious increase in the Raman peak intensity at 1616 cm^−1^ on the test line but a decrease in the control line. The calibration curve was constructed from the standard relative Raman intensity ratios (ITL/ICL). The Raman mapping strategy has found widespread use for signal averaging in SERS LFIA [19,22,23,30,36,38,45,48,61,62]. It should be noted that, in all studies, a strong variation in the Raman signal from point to point within one zone was observed. This effect can be attributed to the roughness of the nitrocellulose membrane and the partial shielding of SERS tags by the membrane, especially for particles located deep inside the membrane. In our recent paper [57], we examined the dependence of SERS signal and mapping uniformity on the density of SERS tags adsorbed in the T zone. This explains the possible origins for the heterogeneous SERS mapping images. To this end, we made SEM imaging of the LFIA strips. In the presence of the target antigen, the SERS tags appear as bright spots in the membrane pores of the SEM image. We found that, in agreement with SERS data, the number of SERS tags gradually decreased with the decrease in analyte concentration, and the detection limit of the SERS LFIA is determined by a lower limit of the particles captured in the test zone.

**Figure 4:**
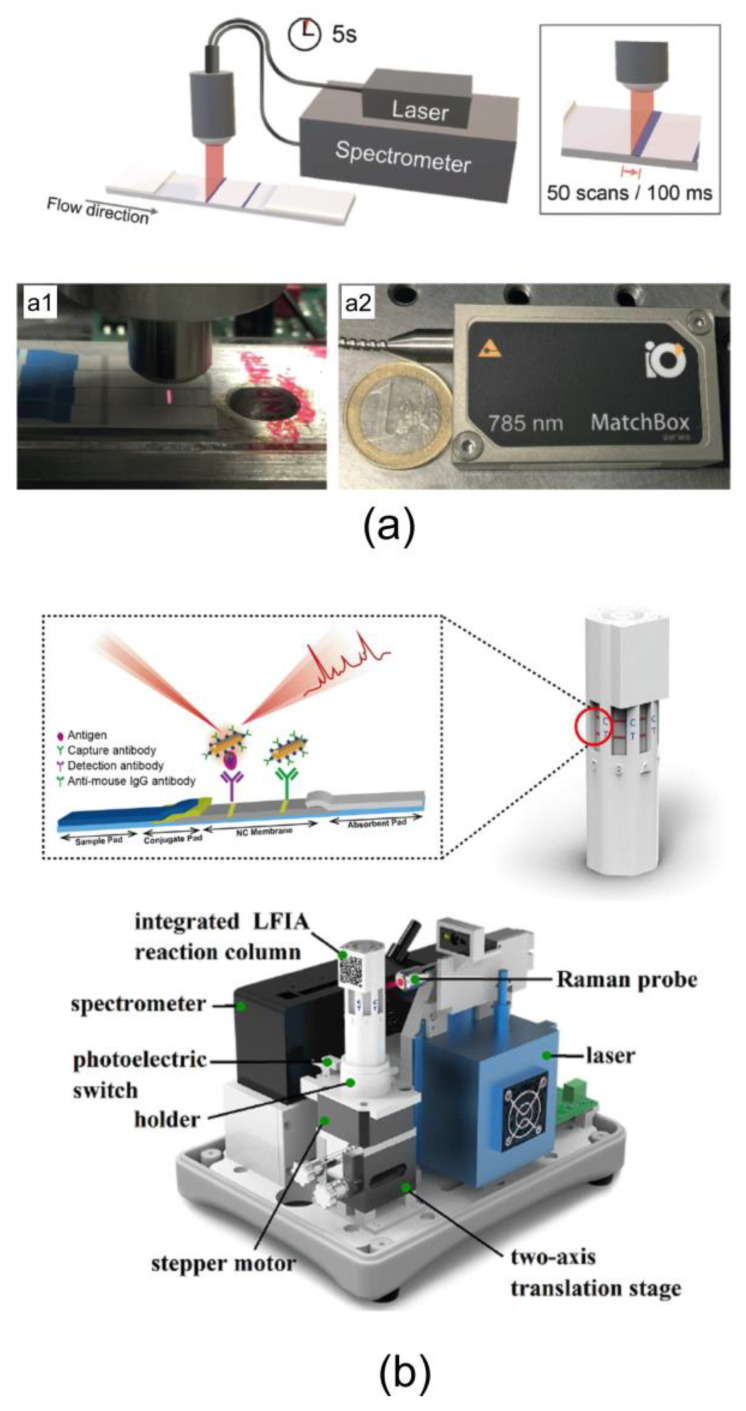
(**a**) Portable Raman/SERS reader with a custom-designed optical fiber probe with laser line focus. Reproduced with permission from [8]. (**b**) Schematic of portable SERS-based LFIA reader. Reproduced with permission from [66].

There are two main problems of current SERS LFIA in the case of using a common Raman microscope as a setup for measurements. First, the measurement is quite time consuming. The Raman mapping of T and C zones on a test strip need to move to the x–y translation stage repeatedly. Therefore, the acquisition of Raman spectra is slow and inefficient. Overall, the acquisition of Raman spectra over hundreds of pixels results in total acquisition times of tens of minutes. Second, Raman microscopes are relatively big and expensive devices, which is why its use leads to the loss of the main advantage of LFIA as a point-of-care method. From this point of view, the construction of simple and compact special reader looks promising for the implementation of SERS LFIA in clinics. Recently, Tran et al. [59] developed a portable SERS reader for rapid scanning of the test strip (Figure 4a).

The main feature of such a device is line illumination along the entire width of the test zone. This illumination is achieved by a custom-designed fiber optical probe with a line focus in combination with a compact diode laser. As a result, such scanning allows for the self-averaging of the SERS signal and reduces the signal accumulation time by up to 5 s. Very recently, Xiao et al. [66] developed a portable and automated SERS-based LFIA reader with an integrated multichannel LFIA reaction column (Figure 4b). The multichannel integrated column is a polygonal columnar structure that can accommodate multiple LFIA strips simultaneously. The device can automatically and point by point detect the Raman signals of multiple points on the T-line (3.8 mm length) with typical integration times of 1 s. The Raman signal intensities of all tested points are averaged through the developed software to obtain a reproducible Raman intensity. At the end of Raman signal acquisition of the channel, the integrated LFIA column rotates automatically to the next detection channel under the drive of the stepper motor. In this way, the SERS signals of the T lines for all strips loaded onto the integrated LFIA column can be measured sequentially and automatically.

Two important notes are presented here. First, the main advantage and clinical rationale of the classical LFIA format is its simplicity for low-cost point-of-care testing. These advantages have already been confirmed during the COVID-19 pandemic, and LFIA is now widely used as a clinical coronavirus antibody test. This, once again, demonstrates the unique capabilities of the LFIA when simplicity and cost of analysis are decisive factors. From this point of view, the SERS-based LFIA, even in portable format, as discussed above, cannot compete with the classic LFIA in terms of simplicity, cost, and ability to conduct analysis at home. The second point concerns the sensitivity and quantification of data. In many clinical scenarios, clinicians would prefer to use more robust methods and quantitative analytical data. For example, in the case of acute myocardial infarction, one needs to assess both absolute concentration of cardiac TrI and its fine variation over observation time. To meet both requirements, one needs a detection sensitivity of about 0.01 ng/L, which is higher than the reported SERS-based data. Recent analysis [11] showed that, in an ideal LFIA format, one analyte molecule delivers just one Au NP to the test zone. In this case, the theoretical LODs were in picograms per milliliter range for a typical LFIA format with 0.1 mL of a 25 kDa analyte. Therefore, SERS-based analytical detection has great potential, but its practical implementation requires additional efforts.

## 4. Limit of Detection for SERS LFIA

The most important quantitative parameter that determines the sensitivity of the immunoassay is the limit of detection (LOD). The LOD is the minimal concentration at which the signal from the analyte is three times higher than the standard deviation for a blank response. For a lateral flow test, LOD is determined by many parameters. The main parameters among them are an affinity of primary and secondary antibodies, a density of antibodies on the tag surface, a motility of tags on the membrane, the optical efficiency of labels, and a level of nonspecific absorption [12]. Thus, it is quite problematic to compare the sensitivity of different LFIA techniques. On the other hand, SERS tags consist of plasmonic nanoparticles, and the construction of the SERS LFIA strip is the same as the typical LFIA strip. The presence of the target molecules can first be identified through a color change in the test zone and can then be quantified by Raman measurement. This makes it possible to compare LODs for conventional optical (including naked eye) and SERS readouts. We evaluate the effectiveness of the use of SERS as a readout method without paying attention to the difference in the design of tests, types of antigens, and antibodies. Table 1 shows the results of determining LOD by colorimetry and SERS in all the articles we analyzed.

Despite the variety of analyzed data, some important patterns can be identified. First, in all studied cases, SERS detection gave a significantly lower LOD compared to the colorimetric method. In more than 95% of cases, the differences in LOD were 10–1000-fold. For antigens of a protein nature (hormones, toxins, and antibodies), for which the sandwich format of immunoassay is used, the most probable LOD is 10 ng/mL for colorimetric detection and 0.1 ng/mL for SERS detection. Thus, SERS LFIA is especially important for those applications in which it is required to detect an antigen with concentrations below 1 ng/mL. An example of such a problem is the detection of cardiac troponin I in the blood plasma of patients with suspected myocardial infarction [70]. Second, we did not find a significant difference in the LOD reduction, depending on the types of SERS tags used. Indeed, a decrease in LOD by three orders of magnitude can be obtained both when using gold nanospheres, which do not greatly enhance Raman scattering, and when using particles with a much larger enhancement factor (nanostars, GERTS, and Au@Ag core/shell particles with embedded Raman reporter). Let us discuss why such a significant gain in sensitivity is obtained. The question of the minimum detectable concentration of the analyte is closely related to the question of the minimum detectable concentration of labels on the membrane. With colorimetric detection, we previously determined the minimum density of spherical gold particles in the test zone, which is necessary in order to distinguish its color from the control [11]. For an average particle size of 60 nm, this surface concentration should be at least 10^6^ mm^−2^. On the other hand, it is well known that using Raman microscopy single SERS tag can be detected even against the background of the signal from the substrate [17]. The level of one SERS tag is obviously a theoretical limit that is unattainable in a real experiment due to the discrete scanning mode. However, it can be assumed that 1000 SERS tags per square millimeter is a detectable value. This gives a 1000-fold reduction in the theoretical detection limit when using SERS LFIA instead of a colorimetric method. Obviously, when detecting such a small number of labels, it is necessary to pay particular attention to two aspects. First, the maximum decrease in nonspecific binding of labels outside the T and C zones. Second, the selection of labels with Raman lines that do not overlap with the background signals from the nitrocellulose membrane and plastic blinking card.

Considering the calibration dependences of the SERS signal intensity on the analyte concentration, one can see that, typically, they represent linear SERS intensity vs. logarithmic concentration scale (Figure 3). In this regard, the calibration points on the concentration scale are most often applied with a 5–10-fold dilution. This means that a huge 10–1000-fold decrease in LOD, in fact, corresponds to one to three sequential dilutions. Therefore, to make the determination of LOD more reliable, one needs additional measurements with more detailed dilutions around the LOD value. Such measurements should provide a linear dependence of the measured SERS intensity at a low analyte concentration close to the LOD.

## 5. Multiplex Biomarker Detection

**Figure 5:**
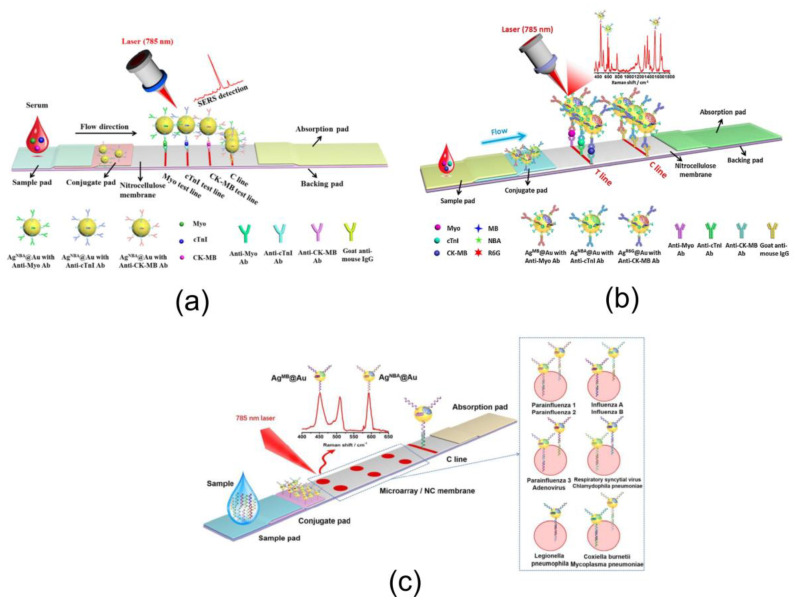
(**a**) Schematic illustration of the multiplex SERS LFA for detection of cardiomarkers using three test zones. Reproduced with permission from [52]. (**b**) Schematic illustration of the multiplex SERS LFA for detection of cardiomarkers using single test zones and three different SERS tags. Reproduced with permission from [53]. (**c**) Schematic illustration of the multiplex SERS LFA for detection of respiratory viruses using multiple test zones and SERS tags. Reproduced with permission from [54].

For many diseases, the detection of a single biomarker is an insufficient diagnosis method because of false positives and negative results [71,72]. Because the simultaneous detection of multiple biomarkers in a single assay can improve diagnosis accuracy, much attention has been paid to multiplex detection [73]. For LFIA, a test strip with multiple test zones for different biomarkers is the most commonly used method for multiplexed detection. In this sense, the SERS-based approach is not different from usual colorimetric detection. For example, Zhang et al. [52] developed a SERS LFIA strip with three test zones for the multiplexed detection of myoglobin (Myo), cardiac troponin I (cTnI), and creatine kinase isoenzyme MB (CKMB), as shown in Figure 5a. Liu et al. [46] used a test strip with two test zones for the simultaneous detection of *Listeria monocytogenes* and *Salmonella enterica* using Au-Ag@MBA SERS tags. The same group developed a strip with two test zones for the multiplex detection of amyloid A and C-reactive with LODs as low as 0.1 and 0.01 ng/mL. Wang et al. [69] designed a test strip with two test zones for the multiplexed detection of Kaposi’s sarcoma and bacillary angiomatosis. Sun et al. [48] used two test line strips for the rapid screening of neuron-specific enolase (NSE) and the S100-b protein.

However, the use of test strips with several test zones does not fully allow SERS tags potential to be realized. Using the fingerprinting nature of Raman spectra, it is possible to design different labels using one type of SERS tag and different reporter molecules. In this case, different recognizing antibodies can be applied in the form of one test zone, and the recognition of different biomarkers can be carried out along different peaks in the Raman spectrum, obtained due to the biospecific adsorption of different SERS tags.

For example, Zhang et al. [52] developed a single T line SERS LFA for quick diagnosis of different cardiomarkers. To this end, three types of Raman-active molecules were embedded inside Au–Ag core–shell SERS tags (Figure 5b). The tags were used as labels for the simultaneous quantification of CK-MB, cTnI, and Myo on a single T line with ultrahigh sensitivity and stability. The reagent consumption and cost as well as preparation time of the SERS LFA were decreased since multiplex assays were integrated on a single line. The LODs for CK-MB, cTnI, and Myo were calculated to be 0.93, 0.89, and 4.2 pg/mL, respectively. Sánchez-Purrà et al. used BPE- and MBA-encoded nanostars as labels for the single test zone simultaneous detection of dengue virus (DENV) and Zika virus (ZIKV). Very recently, Zhang et al. [54] developed a combined multi-tag and multi-T-zone strategy for the simultaneous detection of respiratory viruses. Core shell SERS nanotags encoded with two Raman reporters were chosen as labels and combined on a microarray immobilized on a nitrocellulose membrane for the rapid quantification of the nucleic acids from eleven respiratory viruses on a single strip (Figure 5c).

## 6. Conclusions

In this article, we highlighted SERS-based signal amplification techniques for lateral-flow immunoassay that have been reported in the past decade. The method is based on the use of SERS tags instead of common colloidal gold as labels followed by Raman reading of the signal from the control and test zones of the LFIA strip. The main types of SERS tags used in LFIA, the devices and algorithms used for signal measurement, averaging and baseline correction, and the possibility of multiplexing have been discussed. The main advantages of SERS-LFIA are a three orders of magnitude decrease in LOD and the possibility of quantitative and multiplex detection of a wide range of analytes (proteins, bacteria, viruses, nucleic acids, heavy metals, antibiotics, etc.). These advantages are achieved due to the much higher optical efficiency of the SERS tags and also due to the slow Raman reading and signal isolation. On the other hand, the need for expensive equipment and the long signal acquisition time significantly limit the spread of SERS-LFIA as a standard immunological technique for point-of-care testing. With the appearance, in recent years, of works devoted to the development of compact SERS readers for LFIA strips, progress has been observed in solving these problems. Taking into account that the method theoretically allows one to obtain a detection threshold at the level of several hundred SERS tags, there are many opportunities for this development to be addressed. The most important problems regarding a further decrease in the limit of detection are the development of a lateral flow strip with low Raman background and the correct method for modifying SERS tags to eliminate nonspecific adsorption in complex systems. In addition, combining SERS-LFIA with biochips to create a portable and ultrasensitive detection platform could be a promising approach for point of care diagnostics.

## Figures and Tables

**Table 1:** Limit of detection (LOD) for colorimetric and surface-enhanced Raman scattering (SERS)-based lateral flow immunoassays (LFIA) readout.

Antigen	SERS Tag	Colorimetric LOD	SERS LFIA LOD	Reference
Cardiac TrI	Au@Au-Ag@NBA	5 ng/mL	0.09 ng/mL	[56]
FluB antigen	Au-satelite@MBA	8 µg/mL	0.0085 µg/mL	[58]
Thyroid-stimulating hormone	AuNP@MGITC	1.5 µIU/mL	0.025 µIU/mL	[19]
HIV-1 DNA	AuNP@MGITC	80 pg/mL	8 pg/mL	[20]
Neuron-specific Enolase	Au nanostar@MBA	50 ng/mL	0.08 ng/mL	[21]
Brombuterol	Au nanoflower@MBA	12 pg/mL	0.15 pg/mL	[42]
PCA3 mimic DNA	AuNP@MGITC	3 pM	3 fM	[63]
Staphylococcal enterotoxin B	Au nanoshells@MGITC	10 ng/mL	0.001 ng/mL	[36]
*E. coli*	AuNP@DNTB	N/A	0.52 CFU/mL	[22]
Human IgM	Au-Ag@DNTB	10 ng/mL	0.1 ng/mL	[45]
Cardiac TrI	GERT@NBT	3 ng/mL	0.1 ng/mL	[61]
Colistin	AuNP@DNTB	10 ng/mL	0.10 ng/mL	[25]
Chromium	AgNP@MBA	10^−4^ M	10^−7^ M	[35]
Bisphenol A	Au nanostar@ATP	1 ppb	0.073 ppb	[39]
*Listeria monocytogenes*, *Salmonella enterica*	Au-Ag@MBA	N/A	19 and 27 CFU/mL	[46]
*Escherichia coli* O157:H7	Au-Ag@MBA	5 × 10^5^ CFU/mL	5 × 10^4^ CFU/mL	[47]
Serum amyloid A, C-reactive protein	Fe3O4-Au nanoshells@DNTB	5 ng/mL, 0.5 ng/mL	0.1 and 0.01 ng/mL	[37]
AFP antigen	AuNr@DNTB	1 ng/mL	0.1 ng/mL	[64]
Recombinant nucleoprotein H1N1	Au nanostars@ATP	67 ng/mL	6.7 ng/mL	[41]
Influenza virus A	AuNP@MGITC	5 × 10^4^ pfu/mL	1.9 × 10^4^ pfu/mL	[65]
C-reactive protein	Au-Ag@DNTB	1 ng/mL	0.01 ng/mL	[27]
Zika and dengue viral biomarkers	Au nanostar@MBA, Au nanostar@MBE	10 ng/mL, 50 ng/mL	0.72 and 7.67 ng/mL	[40]
Wild-type pseudorabies virus	Au-Ag@ATP	81 ng/mL	5 ng/mL	[55]
Neomycin	AuNP@ATP	N/A	0.216 pg/m	[67]
Listeria	commercial SERS-S440 Nanotags	6 × 10^7^ pfu/mL	6 × 10^6^ pfu/mL	[28]
NSE and S100-b stroke biomarkers	Au-Ag@MBA, Au-Ag@NBA	N/A	0.01 and 0.05 ng/mL	[48]
Human chorionic gonadotropin	Au-satelite@NT	25 mIU/mL	1.6 mIU/mL	[59]
H1N1 and HAdV viruses	Fe3O4-Ag nanoshells@DNTB	10^4^ and 10^3^ pfu/mL	50 and 10 pfu/mL	[38]
Kaposi’s sarcoma-associated herpesvirus and bacillary angiomatosis	AuNP@MGITC	10 pM	0.043 and 0.074 pM	[68]
Interleukin-6	AuNP@DNTB	5 ng/mL	5 pg/mL	[29]
Stroke biomarker S100-β	Au nanoshells@DNTB	50 pg/mL	5 pg/mL	[30]
aflatoxin M1	Au-Ag@DNTB	N/A	1.7 pg/mL	[49]
*Y. pestis*, *F. tularensis*, and *B. anthracis*	AuNP@MGITC	10,000 CFU/mL	43.4, 45.8, and 357 CFU/mL	[31]
β-conglycinin	AuNP@ATP	1 µg/mL	32 ng/mL	[69]
Influenza A (H7N9)	Au-Ag@ATP	0.08 hemagglutinating units	0.0018 hemagglutinating units	[50]
Human chorionic gonadotropin	GERT@BDT	25 mIU/mL	0.7 mIU/mL	[62]
Myo, cTnI, and CK-MB	Ag-Au@NBA	1 ng/mL	3.2, 0.44, and 0.55 pg/mL	[52]
CK-MB, cTnI, and Myo	Ag-Au@NBA, Ag-Au@R6G Ag-Au@MB	N/A	0.93, 0.89, and 4.2 pg/mL	[53]
Different respiratory viruses	Au-Ag@NBA	1 pM	0.030–0.041 pM	[54]
AFP, CEA, and PSA	AuNR@DTNB	10 ng/mL	0.01 ng/mL	[66]

## References

[1] Osikowicz G., Beggs M., Brookhart P., Caplan D., Ching S., Eck P., Gordon J., Richerson R., Sampedro S., Stimpson D. (1990). One-step chromatographic immunoassay for qualitative determination of choriogonadotropin in urine. Clin. Chem..

[2] Verheijen R., Stouten P., Cazemier G., Haasnoot W. (1998). Development of a one step strip test for the detection of sulfadimidine residues. Analyst.

[3] Kim H., Chung D.R., Kang M. (2019). A new point-of-care test for diagnosis of infectious diseases based on multiplex lateral flow immunoassay. Analyst.

[4] Guo J., Chen S., Guo J., Ma X. (2020). Nanomaterial labels in lateral flow immunoassays for point-of-care-testing. J. Mater. Sci. Technol..

[5] Li F., You M., Li S., Hue J., Liu C., Gonge Y., Yang H., Xu F. (2020). Paper-based point-of-care immunoassays: Recent advances and emerging trends. Biotechnol. Adv..

[6] Bishop J.D., Hsieh H.V., Gasperino D.J., Weig B.H. (2019). Sensitivity enhancement in lateral flow assays: A systems perspective. Lab Chip.

[7] Hristov D.R., Rodriguez-Quijada C., Gomez-Marquez J., Hamad-Schifferli K. (2019). Designing paper-based immunoassays for biomedical applications. Sensors.

[8] Ye H., Liu Y., Zhan L., Liu Y., Qin Z. (2020). Signal amplification and quantification on lateral flow assays by laser excitation of plasmonic nanomaterials. Theranostics.

[9] Dykman L., Khlebtsov N. (2012). Gold nanoparticles in biomedical applications: Recent advances and perspectives. Chem. Soc. Rev..

[10] Wang P., Lin Z.H., Su X., Tang Z.H. (2017). Application of Au based nanomaterials in analytical science. Nano Today.

[11] Khlebtsov B.N., Tumskiy R.S., Burov A.M., Pylaev T.E., Khlebtsov N.G. (2019). Quantification of nanoparticle numbers in the test zone of lateral flow immunoassay strips. ACS Appl. Nano Mater..

[12] Urusov A.E., Zherdev A.V., Dzantiev B.B. (2019). Towards lateral flow quantitative assays: Detection approaches. Biosensors.

[13] Nelis J.L.D., Tsagkaris A.S., Dillon M.J., Hajslova J., Elliott C.T. (2020). Smartphone-based optical assays in the food safety field. TrAC Trends Anal. Chem..

[14] Li X., Yang F., Wong J.X., Yu H.-Z. (2017). Integrated smartphone-app-chip system for on-site parts-per-billion-level colorimetric quantitation of aflatoxins. Anal. Chem..

[15] Wang Y., Yan B., Chen L. (2013). SERS Tags: Novel optical nanoprobes for bioanalysis. Chem. Rev..

[16] Wang Z.H., Zong S.H., Wu l., Zhu D., Cui Y. (2017). SERS-activated platforms for immunoassay: Probes, encoding methods, and applications. Chem. Rev..

[17] Khlebtsov B.N., Burov A.M., Bratashov D.N., Tumskiy R.S., Khlebtsov N.G. (2020). Petal-like gap-enhanced Raman tags with controllable structures for high-speed Raman imaging. Langmuir.

[18] Doering W.E., Piotti M.E., Natan M.J., Freeman R.G. (2007). SERS as a foundation for nanoscale, optically detected biological labels. Adv. Mater..

[19] Choi S., Hwang J., Lee S., Lim D.W., Joo H., Choo J. (2017). Quantitative analysis of thyroid-stimulating hormone (TSH) using SERS-based lateral flow immunoassay. Sens. Actuat. B Chem..

[20] Fu X., Cheng Z., Yu J., Choo P., Chen L., Choo J. (2016). A SERS-based lateral flow assay biosensor for highly sensitive detection of HIV-1DNA. Biosens. Bioelectron..

[21] Gao X., Zheng P., Kasani S., Wu S., Yang F., Lewis S., Nayeem S., Engler-Chiurazzi E., Wigginton J., Simpkins J.W. (2017). Paper-based surface-enhanced Raman scattering lateral flow strip for detection of neuron-specific enolase in blood plasma. Anal. Chem..

[22] Ilhan H., Guven B., Dogan U., Torul H., Evran S., Çetin D., Suludere Z., Saglam N., Boyaci I.H., Tamer U. (2019). The coupling of immunomagnetic enrichment of bacteria with paper-based platform. Talanta.

[23] Jeon J., Lee S.H., Joung Y., Kim K., Choi N., Choo J. (2020). Improvement of reproducibility and thermal stability of surface-enhanced Raman scattering-based lateral flow assay strips using silica-encapsulated gold nanoparticles. Sens. Actuat. B Chem..

[24] Lee S.H., Hwang J., Kim K., Jeon J., Lee S., Ko J., Lee J., Kang M., Chung D.R., Choo J. (2019). Quantitative serodiagnosis of scrub typhus using surface-enhanced Raman scattering-based lateral flow assay platforms. Anal. Chem..

[25] Li Y., Tang S., Zhang W., Cui X., Zhang Y., Jin Y., Zhang X., Chen Y. (2019). A surface-enhanced Raman scattering-based lateral flow immunosensor for colistin in raw milk. Sens. Actuat. B Chem..

[26] Pissuwan D., Gazzana C., Mongkolsuk S., Cortie M.B. (2020). Single and multiple detections of foodborne pathogens by gold nanoparticle assays. WIREs Nanomed. Nanobiotechnol..

[27] Rong Z., Xiao R., Xing S., Xiong G., Yu Z., Wang L., Jia X., Wang K., Wang S., Cong Y. (2018). SERS-based lateral flow assay for quantitative detection of C-reactive protein as an early bio-indicator of radiation-induced inflammatory response in nonhuman primates. Analyst.

[28] Stambach N.R., Carr S.A., Cox C.R., Voorhees K.J. (2015). Rapid detection of listeria by bacteriophage amplification and sers-lateral flow immunochromatography. Viruses.

[29] Wang Y., Sun J., Hou Y., Zhang C., Li D., Li H., Yang M., Fan C., Sun B. (2019). A SERS-based lateral flow assay biosensor for quantitative and ultrasensitive detection of interleukin-6 in unprocessed whole blood. Biosens. Bioelectron..

[30] Wang Y., Hou Y., Li H., Yang M., Zhao P., Sun B. (2019). A SERS-based lateral flow assay for the stroke biomarker S100-β. Microchim. Acta.

[31] Wang R., Kim K., Choi N., Wang X., Lee J., Jeon J.H., Rhie G., Choo J. (2018). Highly sensitive detection of high-risk bacterial pathogens using SERS-based lateral flow assay strips. Sens. Actuat. B Chem..

[32] Wu Z. (2019). Simultaneous detection of listeria monocytogenes and salmonella typhimurium by a sers-based lateral flow immunochromatographic assay. Food Analyt. Meth..

[33] Dzantiev B.B., Byzova N.A., Urusov A.E., Zherdev A.V. (2014). Immunochromatographic methods in food analysis (Review). TrAC Trends Anal. Chem..

[34] Khlebtsov B.N., Bratashov D.N., Khlebtsov N.G. (2018). Tip-functionalized au@ag nanorods as ultrabright surface-enhanced Raman scattering probes for bioimaging in off-resonance mode. J. Phys. Chem. C.

[35] Liang J., Liu H., Lan C., Fu Q., Huang C., Luo Z., Jiang T., Tang Y. (2014). Silver nanoparticle enhanced Raman scattering-based lateral flow immunoassays for ultra-sensitive detection of the heavymetal chromium. Nanotechnology.

[36] Hwang J., Lee S., Choo J. (2016). Application of a sers-based lateral flow immunoassay strip for rapid and sensitive detection of staphylococcal enterotoxin B. Nanoscale.

[37] Liu X., Yang X., Li K., Liu H., Xiao R., Wang W., Wang C., Wang S. (2020). Fe_3_O_4_@Au SERS tags-based lateral flow assay for simultaneous detection of serum amyloid A and C-reactive protein in unprocessed blood sample. Sens. Actuat. B. Chem..

[38] Wang C., Wang C., Wang X., Wang K., Zhu Y., Rong Z., Wang W., Xiao R., Wang S. (2019). Magnetic SERS strip for sensitive and simultaneous detection of respiratory viruses. ACS Appl. Mater. Interfaces.

[39] Lin L.-K., Stanciu L.A. (2018). Bisphenol A detection using gold nanostars in a surface-enhanced Raman scattering improved lateral flow immunochromatographic assay. Sens. Act. B Chem..

[40] Sánchez-Purrà M., Carré-Camps M., de Puig H., Bosch I., Gehrke L., Hamad-Schiffer K. (2017). Surface-enhanced Raman spectroscopy-based sandwich immunoassays for multiplexed detection of zika and dengue viral biomarkers. ACS Infect. Dis..

[41] Maneeprakorn W., Bamrungsap S., Apiwat C., Wiriyachaiporn N. (2016). Surface-enhanced Raman scattering based lateral flow immunochromatographic assay for sensitive influenza detection. RSC Adv..

[42] Fu X., Chu Y., Zhao K., Li J., Deng A. (2017). Ultrasensitive detection of the β-adrenergic agonist brombuterol by a SERS-based lateral flow immunochromatographic assay using flower-like gold-silver core-shell nanoparticles. Microchim. Acta.

[43] Ma Y., Liu H., Chen Y., Gu C., Wei G., Jiang T. (2020). Improved lateral flow strip based on hydrophilic-hydrophobic SERS substrate for ultra-sensitive and quantitative immunoassay. Appl. Surf. Sci..

[44] He D., Wu Z., Cui B., Xu E., Jin Z. (2019). Establishment of a dual mode immunochromatographic assay for Campylobacter jejuni detection. Food Chem..

[45] Jia X., Wang C., Rong Z., Li J., Wang K., Qie Z., Xiao R., Wang S. (2018). Dual dye-loaded Au@Ag coupled to a lateral flow immunoassay for the accurate and sensitive detection of Mycoplasma pneumoniae infection. RSC Adv..

[46] Liu H.-B., Du X.-J., Zang Y.-X., Li P., Wang S. (2017). SERS-based lateral flow strip biosensor for simultaneous detection of listeria monocytogenes and salmonella enterica serotype enteritidis. J. Agric. Food Chem..

[47] Liu H.-B., Chen C.-Y., Zhang C.-N., Du X.-J., Li P., Wang S.H. (2019). Functionalized AuMBA@Ag nanoparticles as an optical and SERS dual probe in a lateral flow strip for the quantitative detection of *Escherichia coli* O157:H7. J. Food Sci..

[48] Sun J., Zhao Y., Hou Y., Li H., Yang M., Wang Y., Sun B. (2019). Multiplexed electrochemical and SERS dual-mode detection of stroke biomarkers: Rapid screening with high sensitivity. New J. Chem..

[49] Wang J., Chen Q., Jin Y., Zhang X., He L., Zhang W., Chen Y. (2020). Surface enhanced Raman scattering-based lateral flow immunosensor for sensitive detection of aflatoxin M1 in urine. Anal. Chim. Acta.

[50] Xiao M., Xie K., Dong X., Wang L., Huang C., Xu F., Xiao W., Jin M., Huang B., Tang Y. (2019). Ultrasensitive detection of avian influenza A (H7N9) virus using surface-enhanced Raman scattering-based lateral flow immunoassay strips. Anal. Chim. Acta.

[51] Zhang W., Tang S., Jin Y., Yang C., He L., Wang J., Chen Y. (2020). Multiplex SERS-based lateral flow immunosensor for the detection of major mycotoxins in maize utilizing dual Raman labels and triple test lines. J. Hazard. Mater..

[52] Zhang D., Huang L., Liu B., Ni H., Sun L., Su E., Chen H., Gu Z., Zhao X. (2018). Quantitative and ultrasensitive detection of multiplex cardiac biomarkers in lateral flow assay with core-shell SERS nanotags. Biosens. Bioelectron..

[53] Zhang D., Huang L., Liu B., Su E., Chen H.-Y., Gu Z., Zhao X. (2018). Quantitative detection of multiplex cardiac biomarkers with encoded SERS nanotags on a single T line in lateral flow assay. Sens. Actuat. B Chem..

[54] Zhang D., Huang L., Liu B., Ge Q., Dong J., Zhao X. (2019). Rapid and ultrasensitive quantification of multiplex respiratory tract infection pathogen via lateral flow microarray based on SERS nanotags. Theranostics.

[55] Shen H., Xie K., Huang L., Wang L., Ye J., Xiao M., Ma L., Jia A., Tang Y. (2019). A novel SERS-based lateral flow assay for differential diagnosis of wildtype pseudorabies virus and gE-deleted vaccine. Sens. Actuat. B Chem..

[56] Bai T., Wang M., Cao M., Zhang J., Zhang K., Zhou P., Liu Z., Liu Y., Guo Z., Lu X. (2018). Functionalized Au@Ag-Au nanoparticles as an optical and SERS dual probe for lateral flow sensing. Anal. Bioanal. Chem..

[57] Khlebtsov B., Khanadeev V., Khlebtsov N. (2016). Surface-enhanced Raman scattering inside Au@Ag core/shell nanorods. Nano Res..

[58] Cho H., Das M., Bhandari P., Irudayaraj J. (2015). High performance immunochromatographic assay combined withsurface enhanced Raman spectroscopy. Sens. Actuators B.

[59] Tran V., Walkenfort B., König M., Salehi M., Schlücker S. (2019). Rapid, quantitative and ultrasensitive POCT: Design of a portable SERS reader for lateral flow assays in clinical chemistry. Angew. Chem..

[60] Lim D.-K., Jeon K.-S., Hwang J.-H., Kim H., Kwon S., Shu Y.D., Nam J.-M. (2011). Highly uniform and reproducible surface-enhanced Raman scattering from dNA-tailorable nanoparticles with 1-nm interior gap. Nat. Nanotechnol..

[61] Khlebtsov B.N., Bratashov D.N., Byzova N.A., Dzantiev B.B., Khlebtsov N.G. (2019). SERS-based lateral flow immunoassay of troponin I by using gap-enhanced Raman tags. Nano Res..

[62] Ye Z., Lin L., Tan Z., Zeng Y.-J., Ruan S., Ye J. (2019). Sub-100 nm multi-shell bimetallic gap-enhanced Raman tags. Appl. Surf. Sci..

[63] Fu X., Wen J., Li J., Lin H., Liu Y., Zhuang X., Tiana C., Chen L. (2019). Highly sensitive detection of prostate cancer specific PCA3 mimic DNA using SERS-based competitive lateral flow assay. Nanoscale.

[64] Lu L., Yu J., Liu X., Yang X., Zhou Z., Jin Q., Xiao R., Wang C. (2020). Rapid, quantitative and ultra-sensitive detection of cancer biomarker by a SERRS-based lateral flow immunoassay using bovine serum albumin coated Au nanorods. RSC Adv..

[65] Park H.J., Yang S.C.H., Choo J. (2016). Early diagnosis of influenza virus a using surface-enhanced Raman scattering-based lateral flow assay. Bull. Korean Chem. Soc..

[66] Xiao R., Lu L., Rong Z., Wang C., Peng Y., Wang F., Wang J., Sun M., Dong J., Wang D. (2020). Portable and multiplexed lateral flow immunoassay reader based on SERS for highly sensitive point-of-care testing. Biosens. Bioelectron..

[67] Shi Q., Huang J., Sun Y., Yin M., Hu M., Hu X. (2018). Utilization of a lateral flow colloidal gold immunoassay strip based on surface-enhanced Raman spectroscopy for ultrasensitive detection of antibiotics in milk. Spectrochim. Acta A Mol. Biomol. Spectrosc..

[68] Wang X., Choi N., Cheng Z., Ko J., Chen L., Choo J. (2017). Simultaneous detection of dual nucleic acids using a SERS-based lateral flow biosensor. Anal. Chem..

[69] Xi J., Yu Q. (2020). The development of lateral flow immunoassay strip tests based on surface enhanced Raman spectroscopy coupled with gold nanoparticles for the rapid detection of soybean allergen β-conglycinin. Spectrochim. Acta A Mol. Biomol. Spectrosc..

[70] Hasić S., Kiseljaković E., Jadrić R., Radovanović J., Winterhalter-Jadrić M. (2003). Cardiac troponin I: The gold standard in acute myocardial infarction diagnosis. Bosnian. J. Basic Med. Sci..

[71] Xu Y., Zhang X., Luan C., Wang H., Chen B., Zhao Y. (2017). Hybrid hydrogel photonic barcodes for multiplex detection of tumor markers. Biosens. Bioelectron..

[72] Zou Z., Yang H., Yan Q., Qi P., Qing Z.H., Zheng J., Xu X., Zhang L., Feng F., Yang R. (2019). Synchronous screening of multiplexed biomarkers of Alzheimer’s disease by a length-encoded aerolysin nanoporeintegrated triple-helix molecular switch. Chem. Commun..

[73] Huang L., Tian S., Zhao W., Liu K., Ma X., Guo J. (2020). Multiplexed Detection of Biomarkers in Lateral-Flow Immunoassays. Analyst.

